# Maternal obesity influences expression and DNA methylation of the adiponectin and leptin systems in human third-trimester placenta

**DOI:** 10.1186/s13148-019-0612-6

**Published:** 2019-02-07

**Authors:** Perrine Nogues, Esther Dos Santos, Hélène Jammes, Paul Berveiller, Lucie Arnould, François Vialard, Marie-Noëlle Dieudonné

**Affiliations:** 1 GIG-EA 7404, Université de Versailles-St Quentin, Université Paris-Saclay, Unité de Formation et de Recherche des Sciences de la Santé Simone Veil, 2 avenue de la Source de la Bièvre, F-78180 Montigny-le-Bretonneux, France; 2 Service de Biologie Médicale, Centre Hospitalier de Poissy-Saint-Germain-en-Laye, Poissy, France; 3 UMR BDR, INRA, ENVA, Université Paris Saclay, Jouy en Josas, France; 4 Service de Gynécologie-Obstétrique, Centre Hospitalier de Poissy-Saint-Germain-en-Laye, Poissy, France; 5 Département de Biologie de la Reproduction, Cytogénétique, Centre Hospitalier de Poissy-Saint-Germain-en-Laye, Poissy, France

**Keywords:** Maternal obesity, Placenta, DNA methylation, Leptin, Adiponectin

## Abstract

**Background:**

It is well established that obesity is associated with dysregulation of the ratio between the two major adipokines leptin and adiponectin. Furthermore, it was recently reported that maternal obesity has a significant impact on placental development. Leptin and adiponectin are present at the fetal-maternal interface and are involved in the development of a functional placenta. However, less is known about leptin and adiponectin’s involvement in the placental alterations described in obese women. Hence, the objective of the present study was to characterize the placental expression and DNA methylation of these two adipokine systems (ligands and receptors) in obese women.

**Results:**

Biopsies were collected from the fetal and maternal sides of third-trimester placenta in obese and non-obese (control) women. In both groups, leptin levels were higher on the fetal side than the maternal side, suggesting that this cytokine has a pivotal role in fetal growth. Secondly, maternal obesity (in the absence of gestational diabetes) was associated with (i) elevated DNA methylation of the leptin promoter on fetal side only, (ii) hypomethylation of the adiponectin promoter on the maternal side only, (iii) significantly low levels of leptin receptor protein (albeit in the absence of differences in mRNA levels and promoter DNA methylation), (iv) significantly low levels of adiponectin receptor 1 mRNA expression on the maternal side only, and (v) elevated DNA methylation of the adiponectin receptor 2 promoter on the maternal side only.

**Conclusion:**

Our present results showed that maternal obesity is associated with the downregulation of both leptin/adiponectin systems in term placenta, and thus a loss of the beneficial effects of these two adipokines on placental development. Maternal obesity was also associated with epigenetic changes in leptin and adiponectin systems; this highlighted the molecular mechanisms involved in the placenta’s adaptation to a harmful maternal environment.

**Electronic supplementary material:**

The online version of this article (10.1186/s13148-019-0612-6) contains supplementary material, which is available to authorized users.

## Background

Obesity is a growing public health problem; it affects almost 30% of women of child-bearing age [1]. In particular, obesity has significant reproductive repercussions during pregnancy. When compared with non-obese women, obese women have a greater likelihood of a poor pregnancy outcome: fetal macrosomia, fetal intrauterine growth restriction (IUGR), preeclampsia, gestational diabetes (GD) mellitus, gestational hypertension, etc. [2]. Several possible mechanistic explanations for the association between obesity and a greater frequency of complications in pregnancy have been proposed. For example, it has been suggested that obesity predisposes women to a harmful, pro-inflammatory environment generated by excess adipose tissue and thus impairs placental functions [3]. It is now well-established that placenta is not just a passive tissue mediating fetal-maternal exchanges; in fact, the placenta can modify its capacity to supply nutrients in response to intrinsic factors (e.g., gestational age) and extrinsic factors (e.g., nutritional and other environmental variations) in the prevailing conditions in utero [4]. Moreover, alterations in placental morphology and functions have been observed in the context of complicated pregnancy. More precisely, excessive accumulation of macrophages and lipids, and high levels of oxidative stress have been observed in placenta sample from obese women. These alterations result in (i) the production of pro-inflammatory cytokines (including interleukin-6, tumor necrosis factor, and monocyte chemotactic protein 1) [5], and (ii) the nitration of several proteins [6]. Uncontrolled placental inflammation and lipotoxicity impairs placental function, with elevated delivery of free fatty acids to the fetal circulation and seem to alter fetal growth and development [3]. Moreover, obesity is also associated with a decrease in the number and activity of the mitochondria in human placental cells [7, 8]. Lastly, abnormal placental angiogenesis and focal immaturity of the villous tree have also been described in women with pregravid obesity [9]. Furthermore, recent studies have clearly demonstrated that placental adaptations to a harmful maternal environment include epigenetic modifications (DNA methylation, DNA hydroxymethylation, histone acetylation, micro-RNAs, etc.) that result in fetal reprogramming during pregnancy [4, 10, 11]. More precisely, the genome regions are differentially methylated and hydroxymethylated: placental DNA methylation levels were 21% higher in an obese group (relative to a non-obese group), whereas hydroxymethylation levels were 31% lower [12].

Adipose tissue is known to synthesize and secrete many different cytokines, including leptin and adiponectin in particular [13]. Both of these adipokines regulate energy homeostasis, inflammation, angiogenesis, and immunomodulation by exerting central or peripheral effects via their specific receptors (i.e., the leptin receptor (LEPR) and adiponectin receptors 1 and 2 (ADIPOR1/R2)) [14, 15]. Furthermore, many studies have clearly described the two adipokines’ involvement in human pregnancy. Leptin and adiponectin have effectively a wide range of important roles, ranging from maternal physiology to embryo implantation and from endo/para/autocrine effects at the fetal-maternal interface to the regulation of conceptus development and fetal growth [16]. More particularly, maternal circulating levels of leptin are elevated during a normal pregnancy because the adipokine is secreted by placental cells. Maternal adiponectin levels increase during the first half of pregnancy and then fall in proportion to the weight gain [17, 18]. In a pregnancy complicated by obesity, circulating leptin concentrations are higher than normal, and circulating adiponectin concentrations are slightly lower than normal throughout pregnancy [19]. Major perturbations of the maternal leptin/adiponectin balance have been described in some pathologies of the female reproductive system, such as GD, pre-eclampsia, and fetal IUGR [20]. Furthermore, we and others have reported that leptin and adiponectin are also secreted respectively by the placenta and the endometrium, as well as by adipose tissue [21, 22]. Both adipokines seemed to favor the development of a functional placenta, with differentiative and anchoring abilities [23, 24]. Indeed, the two cytokines stimulated (i) the molecular and morphological differentiation of cytotrophoblasts into syncytiotrophoblast and (ii) trophoblast invasion from first-trimester placenta [25, 26]. Leptin and adiponectin are also involved in the regulation of immunotolerance and inflammatory processes in the placenta [16]. In fact, the two adipokines exert proinflammatory effects in third-trimester placenta. Leptin appears to be involved in the innate and adaptive immunity of placental cells because it induces the expression of human leucocyte antigen G, the well-known immunosuppressive factor, by trophoblasts [27]. Lastly, leptin and adiponectin exert opposing effects on placental amino acid uptake at term, suggesting that the two adipokines control nutrient delivery to the fetus via the placenta [28]. However, most studies of the leptin and adiponectin systems in human placenta have been performed in non-obese women. Consequently, little is known about the placental expression of leptin or adiponectin in obese women. Hence, in order to better understand the molecular mechanisms underlying placental adaptation in response to maternal obesity, the objective of the present study was to measure the mRNA and protein expression of the leptin/adiponectin systems (ligands and receptors) and epigenetic modifications (DNA methylation) in third-trimester placenta biopsies obtained from a group of obese women and a group of non-obese women.

## Results

### Maternal and neonatal clinical characteristics

Between January 2017 and July 2018, 30 women (age range 27–37) met all the inclusion criteria (see the “Methods” section) and were included in the study (Table 1). All had a singleton pregnancy, and there were no complications of pregnancy. The body mass index (BMI) criterion enabled us to define a control group (BMI = 21.2 ± 3.2 kg/m^2^) and an obese group (BMI = 35.8 ± 4.2 kg/m^2^; *p* <  0.0001 vs. the control group). The study population was homogeneous with regard to demographic parameters, since there were no significant intergroup differences in ethnicity, maternal age, maternal height, or gestational age at delivery. Furthermore, placental characteristics (weight, length, width, and thickness) were similar in the control and obese groups. In addition, we did not observe significant intergroup differences in neonatal characteristics (birth weight, placental weight, and the ratio birth weight/placental weight). Furthermore, a sex-specific analysis did not reveal any male vs. female differences in the neonatal parameters (data not shown). In summary, the control and obese groups did not differ markedly with regard to their placental and neonatal characteristics.

**Table 1:** Maternal and neonatal clinical characteristics

Variable	Control (*N* = 18)	Obese (*N* = 12)	*p* value
Ethnicity	Caucasian	Caucasian	
Maternal age (years)	32 ± 3	34 ± 4	0.13
Maternal height (m)	1.6 ± 0.09	1.6 ± 0.04	0.42
Maternal weight (before pregnancy) (kg)	51.3 ± 10	98.5 ± 13	< 0.0001
BMI (before pregnancy) (kg/m^2^)	21.2 ± 3.2	35.8 ± 4.2	< 0.0001
Gestational age at delivery (weeks of amenorrhea)	39 ± 1.1	38 ± 1	0.085
Placental weight (g)	669.5 ± 135.2	714.8 ± 183.1	0.27
Placental length (cm)	19.3 ± 1.9	18.6 ± 2.3	0.32
Placental width (cm)	16.7 ± 1.6	17.3 ± 1.2	0.23
Placental thickness (cm)	2.1 ± 0.8	2.7 ± 1.1	0.21
Infant’s gender (% male)	50	42	0.34
Birthweight of male infants (g)	3375.56 ± 298.22	3282 ± 165.4	0.26
Birthweight of female infants (g)	3415.6 ± 541.6	3459.6 ± 418.3	0.50
Placental weight for male infants (g)	680.8 ± 132.8	670.1 ± 88.2	0.50
Placental weight for female infants (g)	653.1 ± 158.8	746.8 ± 231.1	0.34
Birthweight/placental weight ratio for male infants	5.4 ± 0.5	4.95 ± 0.52	0.09
Birthweight/placental weight ratio for female infants	5.3 ± 0.75	4.9 ± 1.2	0.20

### Association between obesity and leptin/leptin receptor expression levels in human third-trimester placenta

**Fig. 1:**
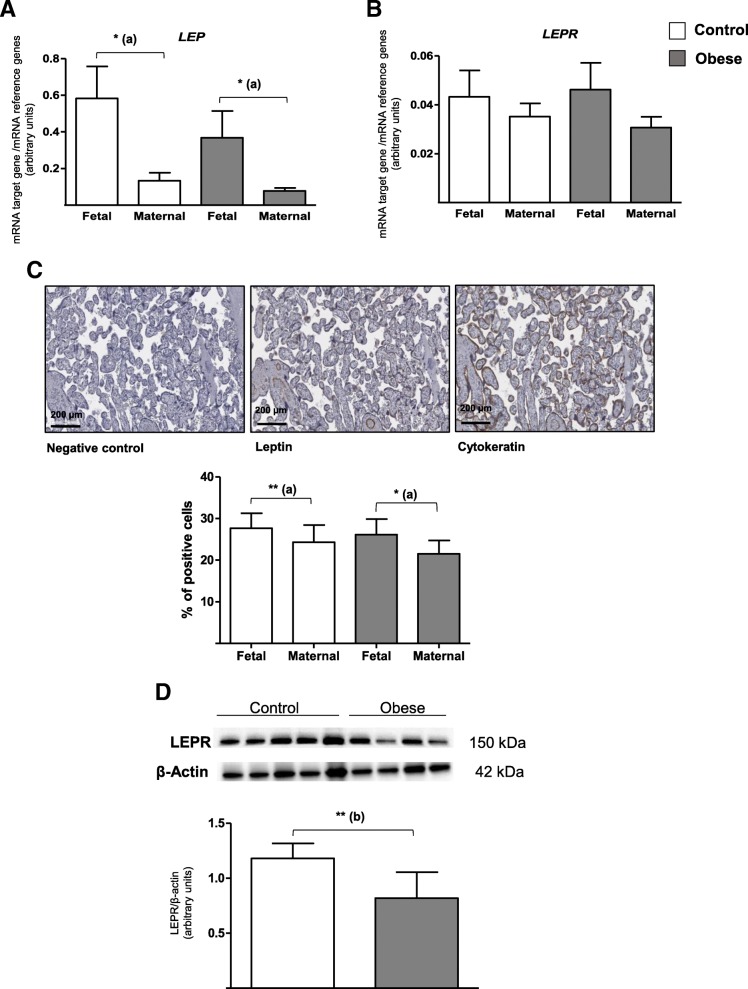
LEP and LEPR expression levels in human third-trimester placental tissue. **a**, **b** mRNA expression of *LEP* and *LEPR*. Total RNA was extracted from the fetal and maternal sides of third-trimester placenta in control and obese women, and then analyzed using RT-qPCR. The data are quoted as the mean ± SEM. *p < 0.05 in a Wilcoxon test. (a) Maternal side vs. fetal side. **c** Leptin protein expression. Third-trimester placental biopsies were fixed, embedded in paraffin, and stained for anti-leptin antibody, as described in the “Methods” section. The data are quoted as the mean ± SEM of *N* = 16 placental sections. *p < 0.05; **p < 0.01 in a Wilcoxon test. (a) Maternal side vs. fetal side. **d** LEPR protein expression. Lysates of third-trimester placental biopsies from control and obese women were extracted and then subjected to Western blot analysis using an anti-LEPR or anti-β-actin antibody, as described in the “Methods” section. The data are representative of five separate experiments and are quoted as the mean ± SEM. **p < 0.01 in a Student *t* test. (b) The obese group vs. the control group

We studied the expression of leptin and its membrane receptors on the fetal and maternal sides of human term placenta in the control and obese groups. Figure 1a shows that leptin gene (*LEP*) expression was higher on the fetal side of the placenta than on the maternal side in both the control and obese groups (by a factor of 4.4 and 5.2, respectively). Moreover, leptin gene expression tended to be lower on both the fetal and maternal sides (by a factor of 0.6 and 0.5, respectively) in placenta from the obese group than in placenta from the control group. The *LEPR* mRNA level was similar on the fetal and maternal sides in both groups (Fig. 1b). Moreover, obesity was not associated with a difference in *LEPR* mRNA expression at fetal and maternal sides of the placenta (Fig. 1b).

We also studied placental protein expression by using immunohistochemistry for leptin and immunoblotting for the LEPR. Semi-quantification immunostaining revealed significantly lower leptin expression on the maternal side (relative to the fetal side) in both control and obese groups (by a factor of 0.9 and 0.8, respectively). These results were consistent with the *LEP* mRNA expression levels (Fig. 1c). Furthermore, leptin protein expression in the placenta was seen to be similar in the obese and control groups. In contrast, LEPR protein expression levels (measured in whole placental tissue) were significantly lower (by a factor of 0.7) in the obese group than in the control group (Fig. 1d).

In summary, leptin expression was significantly lower on the maternal side of the placenta than on fetal side in both the obese and control groups. Maternal obesity did not seem to affect mRNA and protein expression of leptin by the placenta but was associated with lower protein expression of the leptin receptor.

### Association between obesity and ADIPOR1/ADIPOR2 expression levels in human third-trimester placenta

**Fig. 2:**
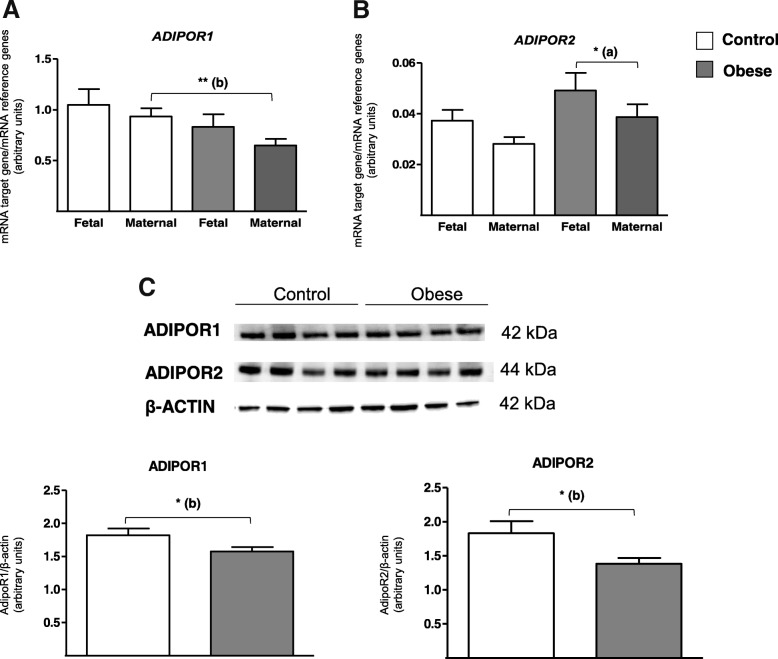
ADIPOR expression in human third-trimester placental tissue. **a**, **b** mRNA expression of *ADIPOR1* and *ADIPOR2*. Total RNA was extracted from the fetal and maternal sides of third-trimester placenta in control and obese women, and then analyzed using RT-qPCR. The data are quoted as the mean ± SEM. *p < 0.05 in a Wilcoxon test. (a) Maternal side vs. fetal side. **p < 0.01 in a Mann-Whitney test. (b) The obese group vs. the control group. **c** ADIPOR1 and ADIPOR2 protein expression. Lysates of third-trimester placental biopsies from control and obese women were extracted and then subjected to Western blot analysis using an anti-ADIPOR1/R2 or anti-β-actin antibody, as described in the “Methods” section. The data are representative of five separate experiments and are quoted as the mean ± SEM. *p < 0.05 in a Student *t* test. (b) The obese group vs. the control group

Since the adiponectin gene (ADIPOQ) is not expressed in placenta, we analyzed the ADIPOR1 and ADIPOR2 system. Figure 2a shows that *ADIPOR1* mRNA expression levels were quite similar on the fetal and maternal sides in both the control and obese groups. However, the *ADIPOR1* mRNA level of the maternal side was significantly lower (by a factor of 0.7) in the obese group than in the control group. As expected, the *ADIPOR2* mRNA level was significantly lower (by a factor of 0.04) than the level of *ADIPOR1* mRNA (*p* <  0.0001, data not shown). *ADIPOR2* mRNA expression was significantly lower (by a factor of 0.8) on the maternal side than on the fetal side in the obese group (Fig. 2b). *ADIPOR2* mRNA level was also lower on the maternal side in the control than in the obese group (by a factor of 0.7) but did not achieve statistical significance. A quantitative immunoblotting analysis of ADIPOR1 and ADIPOR2 revealed a lower protein expression in the obese group than in the control group, by a factor of 0.8 and 0.7 for ADIPOR1 and ADIPOR2, respectively.

### Association between obesity and DNA methylation of leptin/leptin receptor gene promoters in human third-trimester placenta

**Fig. 3:**
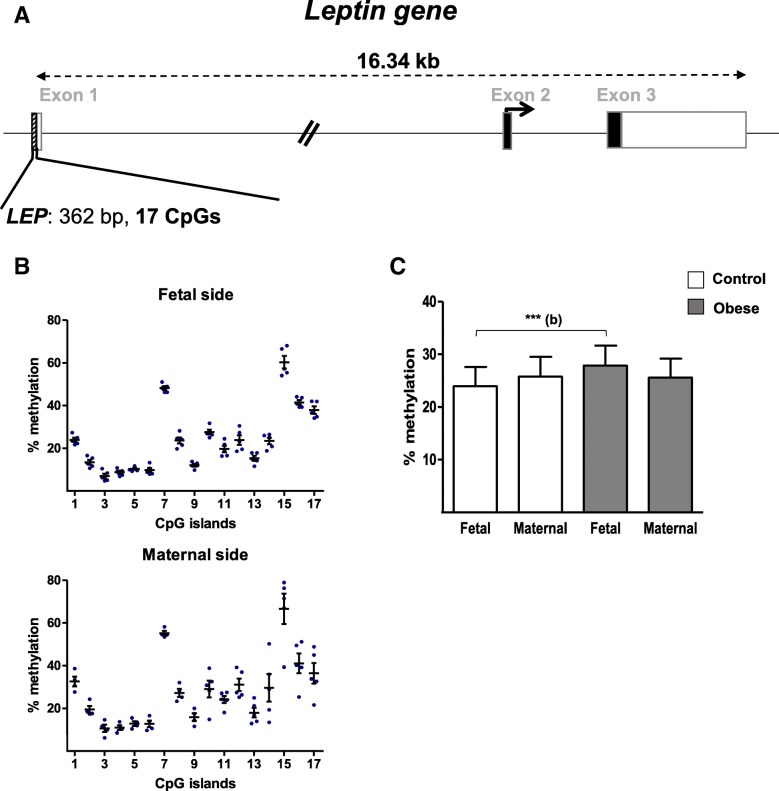
DNA methylation in the promoter region of the *LEP* gene. **a** A schematic representation of the leptin gene, including the CpG islands in the promoter region. **b** The methylation pattern in the *LEP* promoter on the fetal and maternal sides of third-trimester placental biopsies from the control group. **c** The % methylation level in the *LEP* promoter region from third-trimester placenta. DNA was extracted from third-trimester placental biopsies (on the fetal and maternal sides) in the control and obese women. After bisulfite treatment, the methylation level was determined by pyrosequencing. The data are quoted as the mean ± SEM. **p < 0.01 in a Friedman test. (b) The obese group vs. the control group

**Fig. 4:**
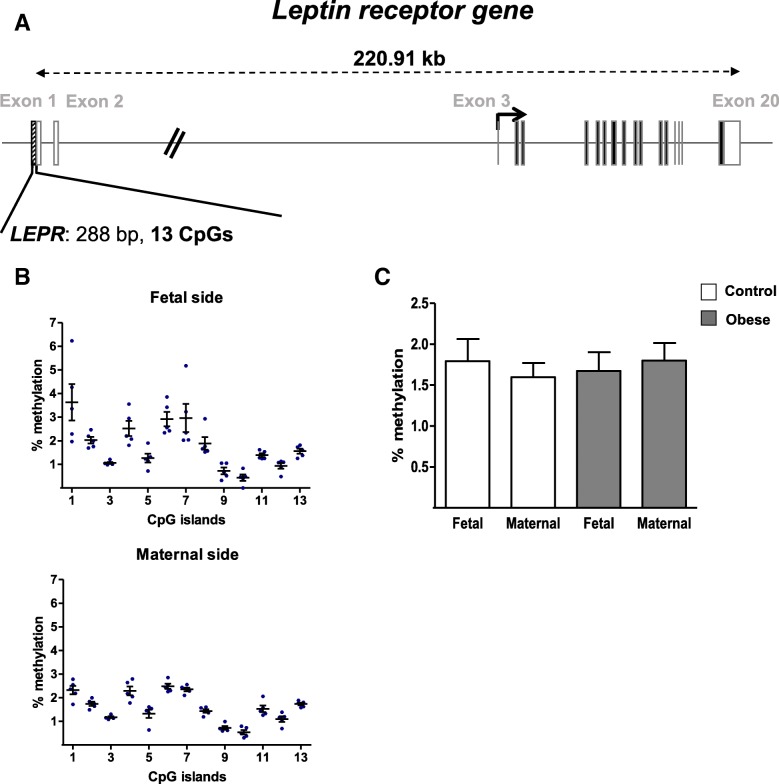
DNA methylation in the promoter region of the *LEPR* gene. **a** A schematic representation of the *LEPR* gene, including the CpG islands in the promoter region. **b** The methylation pattern in the *LEPR* promoter on the fetal and maternal sides of third-trimester placental biopsies from the control group. **c** The % methylation level in the *LEPR* promoter region from third-trimester placenta. DNA was extracted from third-trimester placental biopsies (on the fetal and maternal sides) in the control and obese women. After bisulfite treatment, the methylation level was determined by pyrosequencing. The data are quoted as the mean ± SEM. Statistical significance was assessed in a Friedman test

We analyzed the level of CpG methylation in the gene promoter regions (362 bp and 17 CpG sites for the *LEP* gene promoter, and 288 bp and 13 CpG sites for the *LEPR* promotor; Figs. 3a and 4a, respectively). As shown in Fig. 3b, the methylation levels at the 17 CpG sites in the *LEP* gene promoter ranged from 10 to 75% in placental samples from the control group. Seven CpG sites (#2, #3, #4, #5, #6, #9, and #13) were hypomethylated (< 20%), and four (#7, #15, #16, and #17) were hypermethylated. We hypothesized that the two domains thus defined might have different regulatory roles. The same profile was found on both sides of the placenta, and there were no significant fetal- vs. maternal-side differences in methylation at the CpG sites analyzed. This was also the case for the mean DNA methylation level of the *LEP* promoter region for both groups. However, the mean DNA methylation level in samples from the fetal side was significantly higher (by a factor of 1.2) in the obese group than in the control group (Fig. 3c).

All 13 CpG sites analyzed in the *LEPR* gene promoter displayed a low level of methylation (< 5%). In addition, there was no difference in methylation profile between the fetal and maternal sides of the placenta in the control group (Fig. 4b). Then, there were no significant differences in mean DNA methylation levels between the two groups and on either side of the placenta (Fig. 4c).

Thus, with regard to the leptin system, maternal obesity appears to be associated solely with elevated DNA methylation of the *LEP* gene promoter in samples from the fetal side of the placenta.

### Association between obesity and DNA methylation of ADIPOQ and ADIPOR1/R2 promoters in human third-trimester placenta

**Fig. 5:**
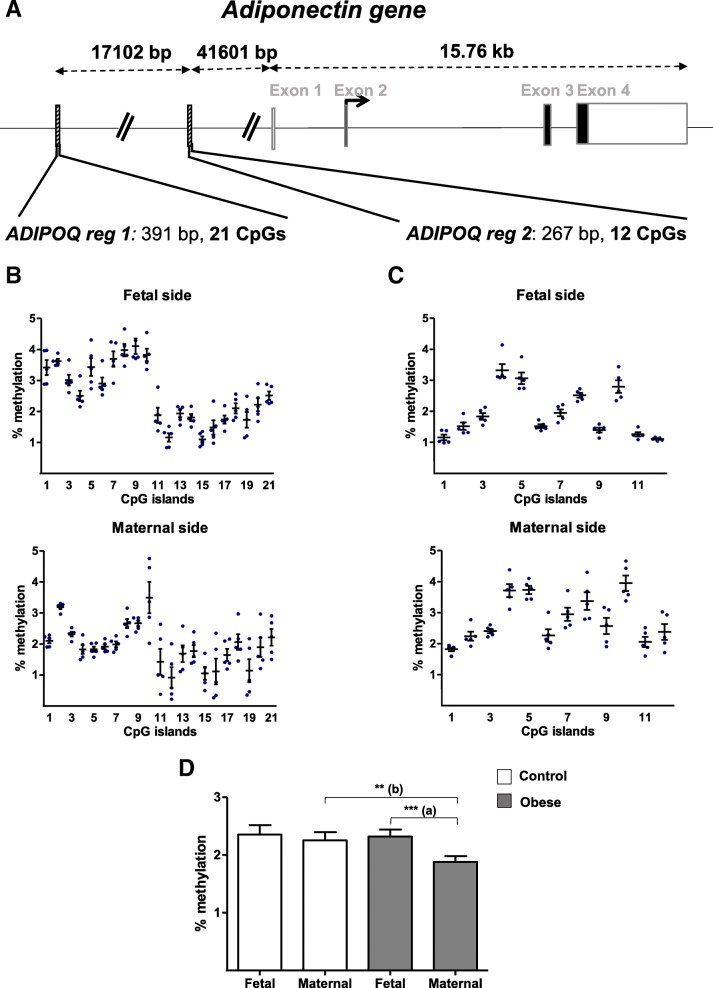
DNA methylation in the promoter regions of the *ADIPOQ* gene. **a** A schematic representation of the *ADIPOQ* gene, including the CpG islands in the promoter region. **b** The methylation pattern in the *ADIPOQ* promoter region 1 (reg 1) on the fetal and maternal sides of third-trimester placental biopsies from the control group. **c** The methylation pattern in the *ADIPOQ* promoter region 2 (reg 2) on the fetal and maternal sides of third-trimester placental biopsies from the control group. **d** The % methylation level in the *ADIPOQ* promoter regions in third-trimester placenta. DNA was extracted from third-trimester placental biopsies (on the fetal and maternal sides) in the control and obese women. After bisulfite treatment, the methylation level was determined by pyrosequencing. The data are quoted as the mean ± SEM. **p < 0.01; ***p < 0.001 in a Friedman test. (a) Maternal side vs. fetal side. (b) The obese group vs. the control group

Two promoter regions of 391 bp (region 1) and of 267 bp (region 2) for *ADIPOQ* were analyzed for DNA methylation at CpG level (21 and 12 CpG sites for region 1 and region 2, respectively) (Fig. 5a). Figure 5b and c shows that all CpG sites analyzed in the two *ADIPOQ* promoter regions were hypomethylated (< 5%). Furthermore, the *ADIPOQ* promoter region 1 could be divided into two domains (CpG sites #1–#10 and #11–#21). The methylation level was significantly higher in the first domain than in the second in the control group (*p* <  0.0020; data not shown). Furthermore, the methylation level for the #1–#10 domains was significantly higher on the fetal side than on the maternal side in the control group (*p* <  0.0020; data not shown). This site-specific higher methylation was also observed for the #11–#21 domains (*p* < 0.0010; data not shown) (Fig. 5b).

In the *ADIPOQ* promoter region 2, the CpG sites #4, #5, and #10 appeared to be less hypomethylated than other CpG sites. Moreover, the region’s methylation level was significantly higher on the maternal side than on the fetal side in the control group (*p* < 0.0409; Fig. 5c). Furthermore, the mean DNA methylation level for the *ADIPOQ* region as a whole was significantly lower (by a factor of 0.8) on the maternal side than on the fetal side in the obese group. The *ADIPOQ* methylation level on placental samples from the maternal side was lower (also by a factor of 0.8) in the obese group than in the control group.

**Fig. 6:**
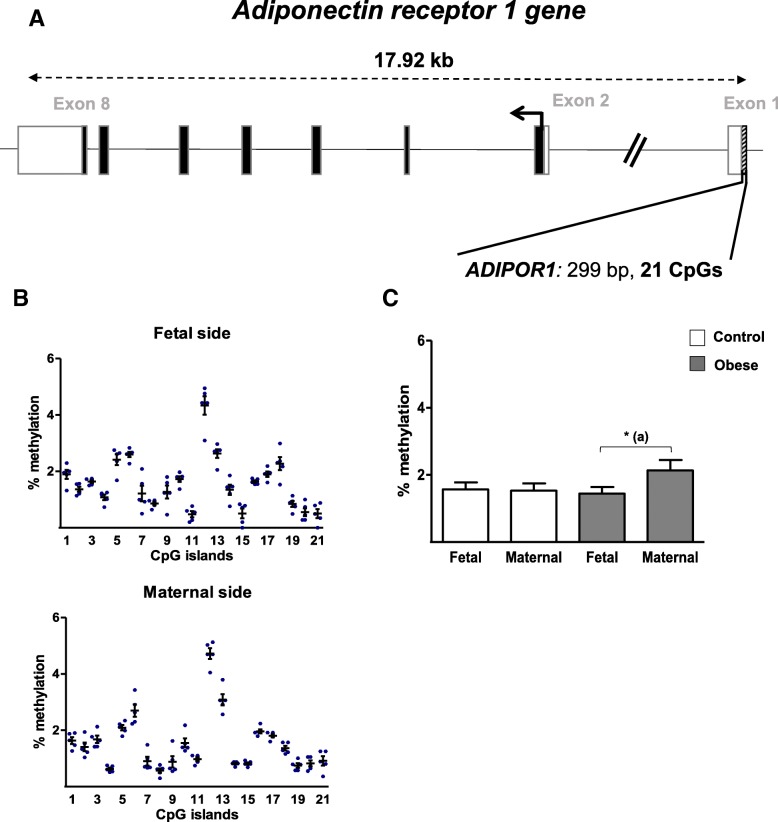
DNA methylation in the promoter region of the *ADIPOR1* gene. **a** A schematic representation of the adiponectin receptor 1 gene (*ADIPOR1*), including the CpG islands in the promoter region. **b** The methylation pattern in the *ADIPOR1* promoter on the fetal and maternal sides of third-trimester placental biopsies from the control group. **c** The % methylation level in the *ADIPOR1* promoter region from third-trimester placenta. DNA was extracted from third-trimester placental biopsies (on the fetal and maternal sides) in the control and obese women. After bisulfite treatment, the methylation level was determined by pyrosequencing. The data are quoted as the mean ± SEM. *p < 0.05 in a Friedman test. (a) Maternal side vs. fetal side

**Fig. 7:**
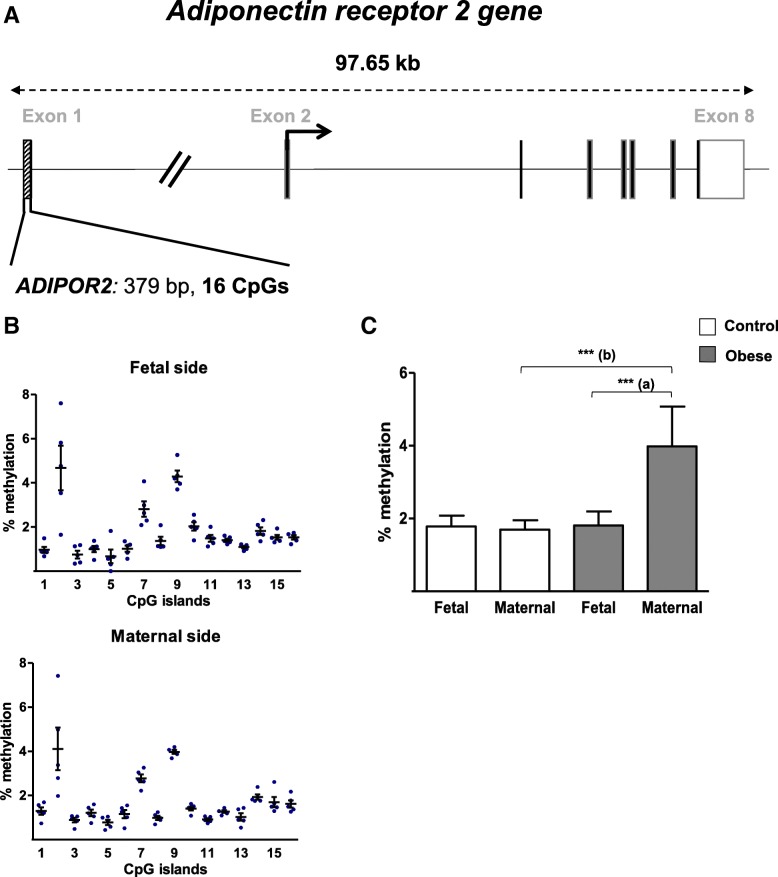
DNA methylation in the promoter region of the *ADIPOR2* gene. **a** A schematic representation of the adiponectin receptor 2 gene (*ADIPOR2*), including the CpG islands in the promoter region. **b** The methylation pattern in the *ADIPOR2* promoter on the fetal and maternal sides of third-trimester placental biopsies from the control group. **c** The % methylation level in the *ADIPOR2* promoter region from third-trimester placenta. DNA was extracted from third-trimester placental biopsies (on the fetal and maternal sides) in the control and obese women. After bisulfite treatment, the methylation level was determined by pyrosequencing. The data are quoted as the mean ± SEM. **p < 0.01; ***p < 0.001 in a Friedman test. (a) Maternal side vs. fetal side. (b) The obese group vs. the control group

Levels of DNA methylation were analyzed in the *ADIPOR1* promoter region (299 bp and 21 CpG sites) and the *ADIPOR2* promoter region (379 bp and 16 CpG sites) (Figs. 6a and 7a). Figures 6b and 7b show that all the analyzed CpG sites in the *ADIPOR1/R2* promoter regions were hypomethylated (< 5%). For *ADIPOR1*, the same methylation profile was seen on both sides of the placenta in the control group and there were no significant methylation differences at each CpG site (Fig. 6b). Figure 6c shows that the mean methylation level was higher (by a factor of 1.5) on the maternal side than on the fetal side in the obese group. For individual CpG sites, the most significant difference was seen for #5, with 2.9-fold increases, on the maternal side than on the fetal side in the obese group (Additional file 1: Figure S1).

For *ADIPOR2*, the same methylation profile was seen on both sides of the placenta in the control group. Again, there were no significant fetal-vs.-maternal side methylation differences at any of the CpG sites (Fig. 7b). Figure 7c shows that the mean methylation level was significantly higher (i) on the maternal side than on the fetal side in the obese women (by a factor of 2.2) and (ii) on the maternal side in the obese group than on the maternal side in the control group (by a factor of 2.4-fold). The elevated methylation level was more prominent for CpG site #2, with a 2.9-fold relative increase on the maternal side than on the fetal side in the obese group, and a 4.8-fold relative increase in the obese group over the control group for the maternal side (Additional file 1: Figure S2).

In summary, maternal obesity appears to be associated with (i) low DNA methylation in the *ADIPOQ* promoter region and (ii) elevated DNA methylation of the *ADIPOR2* promoter region in samples taken from the maternal side of the placenta.

## Discussion

The intrauterine period is critical because it is thought to influence the long-term programming of energy homeostasis regulation and consequently has an important role in determining a child’s susceptibility to obesity and type 2 diabetes [29, 30]. However, the molecular mechanisms linking fetal life to the potential long-term risk of developing metabolic diseases are poorly understood. Epigenetic changes in the placental (such as DNA methylation) might be involved in this link. Indeed, a large body of literature data clearly shows that when confronted with a harmful maternal environment (such as maternal obesity), the placenta can adapt by modifying its own epigenetic programming [12, 31]. Thus, the placenta is a key regulator of fetal growth and development; it not only relays the maternal metabolic environment to the fetus but can also become both a target and source of pathogenic factors affecting the fetus itself [32]. Most studies of the impact of maternal obesity on placental and fetal development have been performed in obese women with GD [33–35]. One of the strengths of our study is its highly characterized cohort of women who are obese but do not suffer from diabetes or cardiovascular disease. Moreover, the ethnic homogeneity of our population helps to avoid discrepancies and possible confounding effects. The homogeneity of the population might also explain why the neonatal and placental parameters in the obese and control groups were homogeneous, relative to other studies. Although obese women may have a normal-sized placenta and give birth to babies with normal birth weight and a normal outcome in the immediate postnatal period, a programming effect on the fetus might reveal itself later in life [3].

Our present study focused on leptin and adiponectin—both adipokines mainly involved in energy metabolism and the regulation of insulin sensitivity [14, 15]. Hence, leptin and adiponectin might be involved in matching the mother’s energy requirements during pregnancy to the growth of the fetoplacental unit [16, 36]. Moreover, elevated leptin levels and, conversely, low adiponectin concentration have been observed in the obese state [18, 19]. In this context, we assessed the mRNA and protein expression levels of adiponectin and leptin systems in human third-trimester placenta of obese and non-obese women, with a novel focus on epigenetic regulation via the DNA methylation of the genes’ respective promoter regions.

Methylation of DNA is the most extensively analyzed epigenetic marker, in view of its fundamental regulatory role in fine-tuning, the gene expression that drives cell lineage and cell fate. During the embryo’s development, the trophoblastic cells acquire a unique DNA methylation profile that clearly differs from that observed in inner mass cells and in other fetal somatic cells [37]. Furthermore, DNA methylation is an attractive biomarker of health; it is relatively stable during sample processing but is sensitive to changes in a tissue’s cellular composition and environment. Furthermore, Wilson et al. recently reviewed the potential of DNA methylation analysis as a placental health biomarker [38]; this emphasizes the value of our study of the methylation status of the *LEP/LEPR*/*ADIPOQ/ADIPOR1/ADIPOR2* promoter regions in a context of maternal obesity.

We first demonstrated that the *LEP* and *ADIPOQ* promoters were moderately methylated (< 70%) in placental samples from our study groups. These results confirm literature reports of the general hypomethylation of placental genes [31]. Low methylation levels in the trophoblast lineage seem to be indispensable for normal extraembryonic development. Moreover, literature reports also served to validate our sample set. Secondly, the present study is the first to have demonstrated that leptin expression is higher on the fetal side of the placenta than the maternal side in both obese and non-obese women. This finding suggests that leptin’s well-known role as a growth factor at the fetal-maternal interface [39, 40] might extend to fetal development. A side-specific expression pattern has already been described for genes reportedly associated with preeclampsia and GD [41]. Thus, our results constitute a new example of side-specific placental expression.

The impact of maternal obesity on placental leptin expression is currently subject to debate. Although most studies did not reveal any BMI-related changes in placental leptin levels [35], a widespread transcriptomic study found that maternal obesity is associated with significantly low placental expression of leptin [42]. In the present study, we showed that maternal obesity is associated with a non-significant downregulation of placental leptin expression. However, our preliminary in vitro experiments revealed that leptin mRNA expression in human syncytiotrophoblast (the main placental cell) isolated from term placenta was significantly lower (by a factor of 0.3) in obese women than in control women (data not shown). Furthermore, we showed that the methylation level of the leptin promoter on the fetal side of the placenta was higher in obese women than in non-obese women. This epigenetic modification was observed in promoter regions close to transcription factor binding sites like SP1, C/EBP, and TATA-box—all of which are known to regulate leptin expression. Hence, our results are in line with other reports whereby obesity might decrease *LEP* promoter activity by decreasing the affinity of some transcription factors for their cognate binding sites [43, 44]. Moreover, Melzner et al. have demonstrated that greater methylation of the CpG sites close to SP1, C/EBP, and TATA-box decreased *LEP* promoter activity in an adipose cell line [45]. Hence, we hypothesize that high levels of leptin promoter methylation in the placenta of obese women might decrease the promoter’s activity and thus result in lower *LEP* mRNA expression. However, our results disagree with those reported by Lesseur et al.; the latter researchers found that the placental DNA methylation of the *LEP* promoter was not elevated in obese women [34]. These discrepancies might be due (at least in part) to the differences in (i) the ethnic and/or clinical characteristics of the study populations and/or (ii) the CpG islands analyzed.

Furthermore, we observed significantly lower placental LEPR protein expression in the obese group than in the non-obese women. This finding is consistent with previous reports and reveals the establishment of an adaptive placental leptin resistance in response to maternal hyperleptinemia [34, 46, 47]. Although we did not observe mRNA regulation or epigenetic modifications of the LEPR promoter, we hypothesize that LEPR protein expression was controlled by a post-transcriptional mechanism—as has already been observed in various tissues and cell types [48, 49]. Our data on the leptin system clearly show low expression of both the ligand and the receptor, which is likely to downregulate leptin’s biological effects in the placenta of obese women.

With regard to adiponectin, we and others have shown that the placenta itself does not express this adipokine [26, 50]. In the present study, we found that the ADIPOQ promoter was hypomethylated in placental samples. This finding suggests that placental adiponectin expression is controlled by other epigenetic modifications (such as short-term, flexible silencing via repressive histone modification, which is important for developmental plasticity), rather than by DNA methylation [11]. The methylation of lysine 27 on histone H3 (H3K27) in human placenta was recently described [51]. Hence, we speculate that H3K27 methylation might be involved in the placental expression of adiponectin. Further experiments are in progress in our laboratory. Lastly, we showed that in human placenta, *ADIPOR2* is 10- to 100-fold less expressed than *ADIPOR1*, as generally described in other tissues and cell types [52, 53]. Interestingly, we observed that maternal obesity is associated with a significantly lower *ADIPOR1 m*RNA expression on the maternal side of the placenta and a significantly lower ADIPOR1 protein expression in the whole placental tissue. Our findings confirm and complete literature data from a general analysis of the human placenta [42]. Furthermore, our present study is the first to have described significantly lower *ADIPOR2* mRNA expression on the maternal side of the placenta than on the fetal side of the placenta in obese women; this may have been due to greater DNA methylation of the *ADIPOR2* promoter. However, the molecular mechanisms underlying the regulation of the *ADIPOR2* promoter have not been characterized. Moreover, we observed lower protein levels of ADIPOR2 in the placenta of obese women, relative to non-obese women. Taken as a whole, our results suggest that these differences in expression may have functional consequences by downregulating the biological signals transmitted by ADIPORs in the placenta of obese women.

## Conclusions

In summary, the leptin and adiponectin systems (i.e., ligands and receptors) are downregulated in the placenta in obese pregnant women, relative to non-obese pregnant women. Given that leptin and adiponectin actively participate in the establishment of morphologically and functionally competent placenta with nutritive, secreting, and anchoring abilities, one can legitimately hypothesize that these placental functions are altered in obese women. Further investigations are underway in our laboratory. Our results also provide a better understanding of the molecular mechanisms (and the epigenetic modifications, more specifically) involved in the placental adaptive response to maternal obesity. Changes in the epigenome early in ontogenesis could be a mechanism of memorization of the maternal obesity, contributing to particular gene expression profiles and thus increase the risk of metabolic syndrome in adulthood.

## Methods

### Materials

The RNA stabilization solution (RNAlater), Pyromark PCR kit, and Pyromark Gold 96 Reagents kit were purchased from Qiagen (Hilden, Germany). The Nucleospin RNA II kit was obtained from Macherey-Nagel (Düren, Germany). Superscript II RNase H-RT, primers, and NovexWedgeWell™ 4–20% Tris-Glycine gel were from Invitrogen (Carlsbad, USA), and RNase inhibitor was obtained from AMRESCO (Solon, USA). The Protein Assay Dye Reagent Concentrate Bradford was purchased from BioRad (München, Germany). Polyclonal goat anti-ADIPOR1 (sc-46749), anti-ADIPOR2 (sc-46756), and mouse anti-leptin (sc-48408) antibodies were from Santa Cruz Biotechnology (Dallas, USA). The rabbit polyclonal anti–LEPR (ab104403) antibody was obtained from Abcam (Cambridge, UK). The mouse monoclonal anti-cytokeratin pan-antibody (KL1) was sourced from Dako Cytomation (Glostrup, Denmark). DyNAmo ColorFlash SYBR Green and the SuperSignal West Pico Chemiluminescent Substrate detection kit were obtained from Fisher Scientific (Waltham, USA). The SPlink Broad kit with DAB chromogen was purchased from GBI Labs Golden Bridge International (Bothell, USA). The Wizard DNA Clean-Up system was bought from Promega (Madison, USA).

### The study population

Women aged between 27 and 37, with a singleton pregnancy, no complications of pregnancy (such as pre-GD and GD), and cesarean delivery were enrolled after the provision of written informed consent. The main inclusion criterion was a first-trimester BMI of 18–25 kg/m^2^ for the non-obese (control) group and 30–40 kg/m^2^ for the obese group. Samples of peripheral blood, cord blood, and placenta were collected from 30 women undergoing elective cesarean delivery at 37 to 41 weeks of amenorrhea. Screening for pre-GD and GD was based on international and French national guidelines. Women with a high risk of GD generally have a personal or family (first-degree) history of diabetes, a history of GD or fetal macrosomia in a previous pregnancy, and a body mass index (BMI) > 25 kg/m^2^, and are aged over 35 [54]. This screening protocol also includes a fasting blood glucose (FBG) assay during the first trimester of pregnancy. Gestational diabetes was defined according to the thresholds published by the International Association of Diabetes and Pregnancy Study Groups [55]. During the first trimester of pregnancy, women with an FBG level of 5.1 mmol/L (92 mg/dL) or more but less than 7.0 mmol/L (126 mg/dL) were considered to have GD. Women with an FBG level of 7.0 mmol/L or more were considered to have preexisting (pre-pregnancy) diabetes. If the FBG level was normal, a 75 g oral glucose tolerance test was then performed at between 24 and 28 weeks. In the test, women with an FBG level of 5.1 mmol/L or more and plasma glucose concentration values of around 10 mmol/L (180 mg/dL) at 1 h and around 8.5 mmol/L (153 mg/dL) at 2 h were also considered to have GD. All women with one or more above-normal measurements were considered to have GD and were treated according to the same protocol. Whenever GD was diagnosed, women were referred to our diabetology department for medical care.

### Placenta tissue sampling

Placenta tissue was sampled within 15 min of cesarean section by experienced clinicians. Tissue biopsies were collected from both the fetal and maternal sides of the placenta. The fetal side consisted of intervillous tissues and chorionic villi. The maternal side consisted mainly of fetal villous tissues but also contained some tissue of maternal origin (decidual basalis). Whenever possible, the analyses were performed using maternal and fetal side samples independently.

### Total RNA extraction and reverse transcription

Human term placental biopsies were collected, placed in RNA stabilization solution, and stored at − 80 °C until use. Around 30 to 40 mg of placental tissue (from the fetal and maternal sides) were disrupted in the lysis buffer from the RNeasy kit. Total RNA was then extracted using the RNeasy kit, according to the manufacturer’s instructions. The quantity and quality of the extracted RNA were measured using an Infinite M200 system (Tecan, Männedorf, Switzerland). Total RNA was stored at − 80 °C until use. Subsequently, 1 μg of total RNA was reverse-transcribed into complementary DNA (cDNA) using random primers. An incubation in the absence of reverse transcriptase was always performed to control for possible contamination by genomic DNA. The cDNA was stored at − 20 °C until use.

### Real-time PCR

Quantitative PCR was performed using a C1000 Thermal Cycler (the CFX96 real-time system; BioRad, Hercules, CA) and the primer sets indicated in Table 2. The second derivative maximum method was used to automatically determine the crossing point (Cp) for individual samples. The three reference genes (coding for ribosomal protein L13A, TATA-binding protein, and B2-microglobulin) had been tested for their stable expression. For each sample, the concentration ratios (target/three reference mRNAs) were calculated using CFX Manager software (version 3.0, BioRad, Hercules, CA) and expressed in arbitrary units. The data were expressed as a percentage of the control value. Calibration curves were log-linear over the quantification range, with correlation coefficients (*r*^2^) > 0.99 and efficiencies ranging from 1.8 to 2. The intra-assay variability of duplicate Cp values never exceeded 0.2 cycles, and the inter-assay variability (coefficient of variation) ranged from 1.9 to 4.1% for 8 to 10 runs of each transcript.

**Table 2:** Primers used for RT-PCR

Primer set	Sequence (5′-3′)	PCR product (bp)
*LEP*	F: TCC ACA CAC GCA GTC AGT CTC R: CTG CCA GTG TCT GGT CCA TC	107
*LEPR*	F: TGG AAG GAG TGG GAA AAC CAA R: TTA AGT CCT TGT GCC CAG GAA	217
*ADIPOR1*	F: TTC TTC CTC ATG GCT GTG ATG T R: AAG AAG CGC TCA GGA ATT CG	71
*ADIPOR2*	F: ATA GGG CAG ATA GGC TGG TTG R: GGA TCC GGG CAG CAT ACA	76
*B2M*	F: TGC TGT CTC CAT GTT TGA TGT ATC T R: TCT CTG CTC CCC ACC TCT AAG T	86
*RP13*	F: CCT GGA GGA GAA GAG GAA AGA GA R: TTG AGG ACC TCT GTG TAT TGT CAA	125
*TBP*	F: TGC ACA GGA GCC AAG AGT GAA R: CAC ACT ACA GCT CCC CAC CA	132

### Immunoblotting

Equal amounts of human term placental biopsies (300 mg) were lysed on ice in buffer containing 20 mM Tris, 137 mM NaCl, 0.2 mM EDTA, 1% Nonidet P40, 1% glycerol, 1 mM sodium orthovanadate, 30 mM beta-glycerophosphate, 100 μg/ml 4-(2-aminoethyl) benzenesulfonyl fluoride hydrochloride, and a cocktail of anti-proteases and anti-phosphatases (5 μg/ml aprotinin, 12.5 μg/ml leupeptin, and 10 mM NaF). Next, the lysates were centrifuged at 12000×*g* for 20 min at 4 °C. The supernatant was collected and the protein concentration was measured in a Bradford assay using bovine serum albumin (BSA) as the standard. The supernatant was then diluted in Laemmli buffer (1:1). Each cell extract (50 μg) was subjected to SDS-PAGE gel electrophoresis (4–20% Tris-Glycine). The proteins were transferred onto a nitrocellulose membrane and blocked in Tris-buffered saline Tween 20 (TBST) 1× buffer (20 mM Tris, 137 mM NaCl, and 0.1% Tween 20) with 2.5% gelatin or 5% BSA for 2 h. Next, the membranes were incubated overnight at room temperature with goat polyclonal anti-AdipoR1 (1:300) or anti-AdipoR2 (1:300) primary antibodies in TBST 1× buffer with 2.5% gelatin or overnight at 4 °C in 5% BSA with rabbit polyclonal anti-LEPR (1:2000). The resulting blots were washed with TBST 1× buffer and incubated with peroxidase-conjugated secondary antibody (1:10000 dilution in TBST 1X buffer) for 1 h at room temperature, and then washed abundantly. Lastly, enhanced chemiluminescence (from the SuperSignal West Pico Chemiluminescent Substrate detection kit) was used for signal detection. To confirm that all samples contained an equal amount of proteins, membranes were reblotted with standard β-actin antibody (1:500) in TBST 1× buffer with 5% milk. The data were analyzed using Rasband WS. ImageJ software (National Institutes of Health, Bethesda, USA).

### Immunohistochemistry

Placentas were sectioned in the vertical plane (from the maternal to the fetal sides), fixed in paraformaldehyde (4%), embedded in paraffin, and cut into 5-μm sections. Deparaffinized sections were heated in 10 mM citrate buffer for 5 min at 160 W in a microwave oven and then left for 20 min at room temperature to heat-induce epitope retrieval. Endogenous peroxidase activity was blocked by incubation with 3% H_2_O_2_, and non-specific IgG binding sites were blocked by incubation with 5% milk for 20 min. The slides were then incubated with primary human anti-leptin antibody (1:50) overnight at 4 °C in PBS with 5% milk. Mouse monoclonal anti-cytokeratin pan-antibody (KL1;1:120) was applied for 20 min at room temperature, in order to characterize trophoblast cells. After extensive washing, the sections were incubated with secondary biotinylated antibody for 20 min at room temperature. Thereafter, peroxidase-conjugated streptavidin was applied for 10 min at room temperature. Visualization was achieved by DAB detection, according to the manufacturer’s instructions. Lastly, slides were counterstained with hematoxylin for 1 min. For negative controls, sections were processed in the absence of primary antibody. Digital images of DAB-stained placenta slides were obtained at a magnification of × 20, using a whole-slide scanner (Aperio ScanScope AT2; Leica Biosystems, Germany). Specific immunostaining was quantified on the maternal and fetal sides separately, using the positive pixel count algorithm in Aperio ImageScope software (Leica Biosystems).

### Extraction of genomic DNA

Around of 100–200 g of placental tissue was used for genomic DNA extraction. The samples were hand-crushed in liquid nitrogen, and then incubated at 55 °C overnight in 10 mM Tris-HCl, pH 7.5, 0.2% SDS, 50 mM NaCl, 10 mM EDTA buffer in the presence of 0.2 mg/ml proteinase K. After incubation in the presence of RNase (final concentration 25 μg/ml) for 60 min at 37 °C and then again in the presence of proteinase K (0.2 mg/ml) for 90 min at 42 °C, the samples were extracted twice with phenol/chloroform (1:1). After centrifugation at 10000×*g* and 4 °C for 5 min, the aqueous phase was collected. Genomic DNA was precipitated in 0.2 M NaCl and 100% ethanol (1:2.5 *v*/*v*) overnight at − 20 °C. After centrifugation (at 10000×*g* and 4 °C for 45 min), the genomic DNA pellet was washed in 70% ethanol, dried, and resuspended in Tris-EDTA buffer (10 mM Tris-HCl, pH 8, 1 mM EDTA). The DNA’s concentration and purity were measured using a Nanodrop 2000 system (Thermo Fisher Scientific, Waltham, USA), and the integrity was checked using agarose gel electrophoresis. The genomic DNA samples were stored at − 20 °C until use.

### Bisulfite conversion of genomic DNA

Genomic DNA samples (1 μg) were submitted to bisulfite conversion, according to a previously described method [56]. Briefly, genomic DNA was denatured for 5 min at 90 °C, and then for 15 min at 37 °C in the presence of a final concentration of 0.2 N NaOH). Bisulfite conversion was performed using fresh bisulfite solution (5 M sodium bisulfite, 130 mM hydroquinone, and 0.35 N NaOH) for 4 h at 55 °C. Bisulfite-converted DNA was purified using the Wizard DNA Clean-Up system, according to the manufacturer’s instructions. The bisulfite-converted DNA was eluted by 40 μl of hot water. The alkali desulphonation was obtained in presence of 0.3 N NaOH (15 min at 37 °C). The bisulfite DNA was precipitated in ammonium acetate and ethanol. After centrifugation (15,000×*g* at 4 °C, for 45 min) and washing, the bisulfite-converted DNA was dissolved in Tris-EDTA buffer, stored at − 20 °C, and analyzed soon afterwards.

### PCR amplification, pyrosequencing, and DNA methylation analysis

Pyrosequencing is a quantitative, real-time sequencing technology that measures the DNA methylation levels (in %) at each CpG site in a given genomic region. Using bisulfite-converted DNA, PCR amplification was performed with specific primers designed using MethylPrimer Express software (version 1.0, Thermo Fisher Scientific). In the present study, we analyzed the promoter regions of the genes coding for leptin (LEP), adiponectin (*ADIPOQ*), and their specific receptors *LEPR*, *ADIPOR1*, and *ADIPOR2*. The regions analyzed were defined according to different criteria: (i) the detection of CpG islands using ENSEMBL (https://www.ensembl.org/index.html), the UCSC Genome Browser (created by the Genome Bioinformatics Group at UC Santa Cruz; http://genome.ucsc.edu/cgi-bin/hgGateway) and the Eukaryotic Promoter Database (created by the Swiss Institute of Bioinformatics; https://epd.vital-it.ch/index.php) and (ii) the presence of CCCTC-binding factor sites using ENSEMBL. CCCTC-binding factor is an important regulatory factor that is sensitive to the DNA methylation level. Moreover, for the chosen leptin and adiponectin promoter regions, we referred to the literature data published by Bouchard et al. [33, 43, 57]. The gene promoter regions and the number of CpG sites analyzed are described in Table 3.

**Table 3:** Selected regions, reference, location, PCR amplification primers, PCR template size, pyrosequencing primers, and number of CpG sites analyzed

Gene symbol Ensembl accession number	Selected regions Start-End	PCR Primer sequences 5′-3′ (bp)	PCR product size (bp)	Pyrosequencing primer sequences 5′- 3′ (bp)	CpG sites included
*Leptin (LEP)* **ENSG00000174697**	Chr7: 128,241,050 Chr7: 128,241,412	F: ATTTTTGGGAGGTATTTAAGGG R: ACATCCCTCCTAACTCAATTT	362	Pyro1: GTTATTTTGAGGGG Pyro2: TTATAAGAGGGG	#17
*Adiponectin (ADIPOQ)* **ENSG00000181092**	Reg1: Chr3: 186783987 Chr3: 186,784,378	F: GGAGGGTTTTAGGTTTTATTTG R: ACCACCTCAACTACACCTTAAA	391	Pyro1: TGGGGAAGGGTTGGAGGTA Pyro2: GTATTGTTGGGGG	#21
	Reg2: Chr3: 186801089 Chr3: 186,801,356	F: GTTTTTTTGGAGAGGAGAGAAAG R: CCRCAAAAAAAACAACTCTC	267	Pyro1: TTTAGGGAGAAAAAGAAGA Pyro2: GTAGTATTAAGAAGGAG	#12
*Leptin receptor (LEPR)* **ENSG00000116678**	Chr1: 65,420,597 Chr1: 65,420,959	F: TTTGGTTTGGGTAGGTTGT R: AAAAAAACCAAAACTCCCC	288	Pyro1: TTTGGTTTGGGTAGGTTGT Pyro2: GTTAAAGGTATAT	#13
*Adiponectin receptor 1 (ADIPOR1)* **ENSG00000159346**	Chr1: 202,957,739 Chr1: 202,958,035	F: TGGTAATTTAATGYGGTTGTT R: CCTAACCTCCAAACATCCA	299	Pyro1: TGGTAATTTAATGYGGTTGTT Pyro2: GTAGTATTTATAGGATTGT	#21
*Adiponectin receptor 2 (ADIPOR2)* **ENSG00000006831**	Chr12: 1,691,140 Chr12: 1,691,555	F: TAGYGGTGGTTTTTAAGAAGT R: AACTAAAACAACTACACCCRAA	379	Pyro1: TAGYGGTGGTTTTTAAGAAGT Pyro2: AGGTGAGAGTTGAGGGG	#16

*F* forward, *R* reverse

Bisulfite-converted DNA (around 50 ng) was amplified in a final 25 μL volume using the Pyromark PCR kit, in accordance with the manufacturer’s recommendations. Next, 20 μL of PCR product was used for pyrosequencing with the Pyromark Gold Q24 Reagents kit and the PyroMark Q24 pyrosequencer, according to the manufacturer’s instructions. For each region, pyrosequencing runs were performed in duplicate. When a duplicate yielded an inconsistent % methylation (more than a 5% difference), the assay was repeated in duplicate. The % methylation per CpG was then obtained by calculating the mean of the replicates that passed the quality control using Pyromark Q24 software (version 1.0, Qiagen, Hilden, Germany).

### Statistical analyses

All values were expressed as the mean ± SEM. For the clinical characteristics, intergroup differences were probed using a Mann-Whitney test. Wilcoxon’s matched-pairs test was used for mRNA expression (to compare placental sides), leptin protein expression, and also for individual CpG values. Mann-Whitney test was used for mRNA expression and individual CpG values to compare control and obese groups. Student *t* test was used for ADIPOR1-R2 and LEPR protein expressions to compare control and obese groups. Friedman’s test was used for DNA methylation analyses. All statistical analyses were performed with GraphPad Prism software (version 5.0, GraphPad, La Jolla, USA).

## Additional file

Additional file 1: **Figure S1.** DNA methylation in the promoter region of the *ADIPOR1* gene in obese placenta. The methylation pattern in the *ADIPOR1* promoter on the fetal and maternal sides of third-trimester placental biopsies from the obese group. The data are quoted as the mean ± SEM. *: *p* < 0.05 for #5 in a Wilcoxon test. Maternal side vs. fetal side in the obese group. Figure S2. DNA methylation in the promoter region of the *ADIPOR2* gene in obese placenta. The methylation pattern in the *ADIPOR1* promoter on the fetal and maternal sides of third-trimester placental biopsies from the obese group. The data are quoted as the mean ± SEM. *: *p* < 0.05 for #2 in a Wilcoxon test on Maternal side vs. fetal side in the obese group. **: *p* < 0.01 for #2 in a Mann-Whitney test. Maternal side in the obese group vs. the control group. (PPTX 180 kb)

## Abbreviations

ADIPOQ: Adiponectin
ADIPOR: Adiponectin receptor
BMI: Body mass index
BSA: Bovine serum albumin
CpG: Cytosine phosphoguanosine
FBG: Fasting blood glucose
GD: Gestational diabetes
IUGR: Intra-uterine growth restriction
LEP: Leptin promoter
LEPR: Leptin receptor promoter
TBST: Tris-buffered saline Tween 20

## References

[1] Kim SY, Dietz PM, England L, Morrow B, Callaghan WM (2007). Trends in pre-pregnancy obesity in nine states, 1993–2003. Obesity.

[2] Jeve YB, Konje JC, Doshani A (2015). Placental dysfunction in obese women and antenatal surveillance strategies. Best Pract Res Clin Obstet Gynaecol.

[3] Myatt L, Maloyan A (2016). Obesity and placental function. Semin Reprod Med.

[4] Sandovici I, Hoelle K, Angiolini E, Constância M (2012). Placental adaptations to the maternal–fetal environment: implications for fetal growth and developmental programming. Reprod BioMed Online.

[5] Challier J, Basu S, Bintein T, Hotmire K, Minium J, Catalano P (2008). Obesity in pregnancy stimulates macrophage accumulation and inflammation in the placenta. Placenta.

[6] Roberts VHJ, Smith J, McLea SA, Heizer AB, Richardson JL, Myatt L. Effect of increasing maternal body mass index on oxidative and nitrative stress in the human placenta. Placenta 2009;30:169–175. doi:10.1016/j.placenta.2008.11.019. 10.1016/j.placenta.2008.11.019 PMC2657925 19100619

[7] Hastie R, Lappas M (2014). The effect of pre-existing maternal obesity and diabetes on placental mitochondrial content and electron transport chain activity. Placenta.

[8] Holland O, Dekker Nitert M, Gallo LA, Vejzovic M, Fisher JJ, Perkins AV. Review: placental mitochondrial function and structure in gestational disorders. Placenta. n.d.. 10.1016/j.placenta.2016.12.012. 10.1016/j.placenta.2016.12.012 28024805

[9] Loardi C, Falchetti M, Prefumo F, Facchetti F, Frusca T (2016). Placental morphology in pregnancies associated with pregravid obesity. J Matern Fetal Neonatal Med.

[10] Elshenawy S, Simmons R (2016). Maternal obesity and prenatal programming. Mol Cell Endocrinol.

[11] Vaiman D (2017). Genes, epigenetics and miRNA regulation in the placenta. Placenta.

[12] Mitsuya K, Parker AN, Liu L, Ruan J, Vissers MCM, Myatt L (2017). Alterations in the placental methylome with maternal obesity and evidence for metabolic regulation. PLoS One.

[13] Kuryszko J, Sławuta P, Sapikowski G (2016). Secretory function of adipose tissue. Pol J Vet Sci.

[14] Li S, Li X (2016). Leptin in normal physiology and leptin resistance. Sci Bull.

[15] Wang ZV, Scherer PE (2016). Adiponectin, the past two decades. J Mol Cell Biol.

[16] Dos SE, Duval F, Vialard F, Dieudonné M-N (2015). The roles of leptin and adiponectin at the fetal-maternal interface in humans. Horm Mol Biol Clin Investig.

[17] Lekva T, Roland MCP, Michelsen AE, Friis CM, Aukrust P, Bollerslev J (2017). Large reduction in adiponectin during pregnancy is associated with large-for-gestational-age newborns. J Clin Endocrinol Metab.

[18] Mitanchez D, Jacqueminet S, Nizard J, Tanguy M-L, Ciangura C, Lacorte J-M, et al. Effect of maternal obesity on birthweight and neonatal fat mass: a prospective clinical trial. PLoS One. 2017;12. 10.1371/journal.pone.0181307. 10.1371/journal.pone.0181307 PMC5531500 28750045

[19] Pagano C, Marin O, Calcagno A, Schiappelli P, Pilon C, Milan G (2005). Increased serum resistin in adults with Prader-Willi syndrome is related to obesity and not to insulin resistance. J Clin Endocrinol Metab.

[20] Miehle K, Stepan H, Fasshauer M (2012). Leptin, adiponectin and other adipokines in gestational diabetes mellitus and pre-eclampsia. Clin Endocrinol.

[21] Henson MC, Swan KF, O’Neil JS (1998). Expression of placental leptin and leptin receptor transcripts in early pregnancy and at term. Obstet Gynecol.

[22] Dos Santos E, Serazin V, Morvan C, Torre A, Wainer R, de Mazancourt P (2012). Adiponectin and leptin systems in human endometrium during window of implantation. Fertil Steril.

[23] Gonzalez RR, Devoto L, Campana A, Bischof P (2001). Effects of leptin, interleukin-1α, interleukin-6, and transforming growth factor-β on markers of trophoblast invasive phenotype. Endocrine.

[24] Benaitreau D, Santos ED, Leneveu M-C, Alfaidy N, Feige J-J, de Mazancourt P (2010). Effects of adiponectin on human trophoblast invasion. J Endocrinol.

[25] Ge YC, Li JN, Ni XT, Guo CM, Wang WS, Duan T (2011). Cross talk between cAMP and p38 MAPK pathways in the induction of leptin by hCG in human placental syncytiotrophoblasts. Reproduction.

[26] Benaitreau D, Santos ED, Leneveu M-C, De Mazancourt P, Pecquery R, Dieudonné M-N (2010). Adiponectin promotes syncytialisation of BeWo cell line and primary trophoblast cells. Reprod Biol Endocrinol RBE.

[27] Barrientos G, Toro A, Moschansky P, Cohen M, Garcia MG, Rose M (2015). Leptin promotes HLA-G expression on placental trophoblasts via the MEK/Erk and PI3K signaling pathways. Placenta.

[28] Hauguel-de Mouzon S, Lepercq J, Catalano P (2006). The known and unknown of leptin in pregnancy. Am J Obstet Gynecol.

[29] Catalano PM (2003). Obesity and pregnancy—the propagation of a viscous cycle?. J Clin Endocrinol Metab.

[30] Catalano PM, McIntyre HD, Cruickshank JK, McCance DR, Dyer AR, Metzger BE (2012). The hyperglycemia and adverse pregnancy outcome study: associations of GDM and obesity with pregnancy outcomes. Diabetes Care.

[31] Nomura Y, Lambertini L, Rialdi A, Lee M, Mystal EY, Grabie M (2014). Global methylation in the placenta and umbilical cord blood from pregnancies with maternal gestational diabetes, preeclampsia, and obesity. Reprod Sci.

[32] Liguori A, D’Armiento FP, Palagiano A, Balestrieri ML, Williams-Ignarro S, de Nigris F (2007). Effect of gestational hypercholesterolaemia on omental vasoreactivity, placental enzyme activity and transplacental passage of normal and oxidised fatty acids. BJOG Int J Obstet Gynaecol.

[33] Bouchard L, Hivert M-F, Guay S-P, St-Pierre J, Perron P, Brisson D (2012). Placental adiponectin gene DNA methylation levels are associated with mothers’ blood glucose concentration. Diabetes.

[34] Lesseur C, Armstrong DA, Paquette AG, Li Z, Padbury JF, Marsit CJ (2014). Maternal obesity and gestational diabetes are associated with placental leptin DNA methylation. Am J Obstet Gynecol.

[35] Martino J, Sebert S, Segura MT, García-Valdés L, Florido J, Padilla MC (2016). Maternal body weight and gestational diabetes differentially influence placental and pregnancy outcomes. J Clin Endocrinol Metab.

[36] Pérez-Pérez A, Toro A, Vilariño-García T, Maymó J, Guadix P, Dueñas JL, et al. Leptin action in normal and pathological pregnancies. J Cell Mol Med 2018:n/a-n/a. doi:10.1111/jcmm.13369. 10.1111/jcmm.13369 PMC5783877 29160594

[37] Senner CE, Krueger F, Oxley D, Andrews S, Hemberger M (2012). DNA methylation profiles define stem cell identity and reveal a tight embryonic-extraembryonic lineage boundary. Stem Cells Dayt Ohio.

[38] Wilson SL, Robinson WP (2018). Utility of DNA methylation to assess placental health. Placenta.

[39] Sagawa N, Yura S, Itoh H, Mise H, Kakui K, Korita D (2002). Role of leptin in pregnancy—a review. Placenta.

[40] Howell KR, Powell TL (2017). Effects of maternal obesity on placental function and fetal development. Reproduction.

[41] Gallo LA, Barrett HL, Dekker NM. Review: placental transport and metabolism of energy substrates in maternal obesity and diabetes. Placenta. n.d.. 10.1016/j.placenta.2016.12.006. 10.1016/j.placenta.2016.12.006 27993398

[42] Saben J, Zhong Y, McKelvey S, Dajani NK, Andres A, Badger TM (2014). A comprehensive analysis of the human placenta transcriptome. Placenta.

[43] Bouchard L, Thibault S, Guay S-P, Santure M, Monpetit A, St-Pierre J (2010). Leptin gene epigenetic adaptation to impaired glucose metabolism during pregnancy. Diabetes Care.

[44] Marchi M, Lisi S, Curcio M, Barbuti S, Piaggi P, Ceccarini G (2011). Human leptin tissue distribution, but not weight loss-dependent change in expression, is associated with methylation of its promoter. Epigenetics.

[45] Melzner I, Scott V, Dorsch K, Fischer P, Wabitsch M, Brüderlein S (2002). Leptin gene expression in human preadipocytes is switched on by maturation-induced demethylation of distinct CpGs in its proximal promoter. J Biol Chem.

[46] Farley DM, Choi J, Dudley DJ, Li C, Jenkins SL, Myatt L (2010). Placental amino acid transport and placental leptin resistance in pregnancies complicated by maternal obesity. Placenta.

[47] Tessier DR, Ferraro ZM, Gruslin A (2013). Role of leptin in pregnancy: consequences of maternal obesity. Placenta.

[48] Good DJ (2000). How tight are your genes? Transcriptional and posttranscriptional regulation of the leptin receptor, NPY, and POMC genes. Horm Behav.

[49] Cammisotto PG, Bendayan M, Levy E (2012). Regulation of leptin receptor expression in human polarized Caco-2/15 cells. Endocr Metab Immune Disord Drug Targets.

[50] Caminos JE, Nogueiras R, Gallego R, Bravo S, Tovar S, García-Caballero T (2005). Expression and regulation of adiponectin and receptor in human and rat placenta. J Clin Endocrinol Metab.

[51] Nelissen ECM, Montfoort V, P.A A, Dumoulin JCM, Evers JLH. Epigenetics and the placenta. Hum Reprod Update 2011;17:397–417. doi:10.1093/humupd/dmq052. 10.1093/humupd/dmq052 20959349

[52] Yamauchi T, Kamon J, Ito Y, Tsuchida A, Yokomizo T, Kita S (2003). Cloning of adiponectin receptors that mediate antidiabetic metabolic effects. Nature.

[53] Husseini A (2010). Expression of adiponectin receptors in human placenta and its possible implication in gestational diabetes. Am J Biochem Biotechnol.

[54] Collège National des Gynécologues et Obstétriciens Français. J Gynécologie Obstétrique Biol Reprod 2005;34:513. doi:10.1016/S0368-2315(05)82867-4.

[55] International Association of Diabetes and Pregnancy Study Groups Recommendations on the Diagnosis and Classification of Hyperglycemia in Pregnancy. Diabetes Care 2010;33:676–682. doi:10.2337/dc09-1848. 10.2337/dc09-1848 PMC2827530 20190296

[56] Kiefer H, Jouneau L, Campion É, Rousseau-Ralliard D, Larcher T, Martin-Magniette M-L (2016). Altered DNA methylation associated with an abnormal liver phenotype in a cattle model with a high incidence of perinatal pathologies. Sci Rep.

[57] Hogg K, Blair JD, von Dadelszen P, Robinson WP (2013). Hypomethylation of the LEP gene in placenta and elevated maternal leptin concentration in early onset pre-eclampsia. Mol Cell Endocrinol.

