# Supplementary figures and images for: Maternal obesity influences expression and DNA methylation of the adiponectin and leptin systems in human third-trimester placenta
Source: Clin Epigenetics. 2019 Feb 7;11:20. doi: 10.1186/s13148-019-0612-6 (PMC6367801; doi:10.1186/s13148-019-0612-6)

## Slide 1
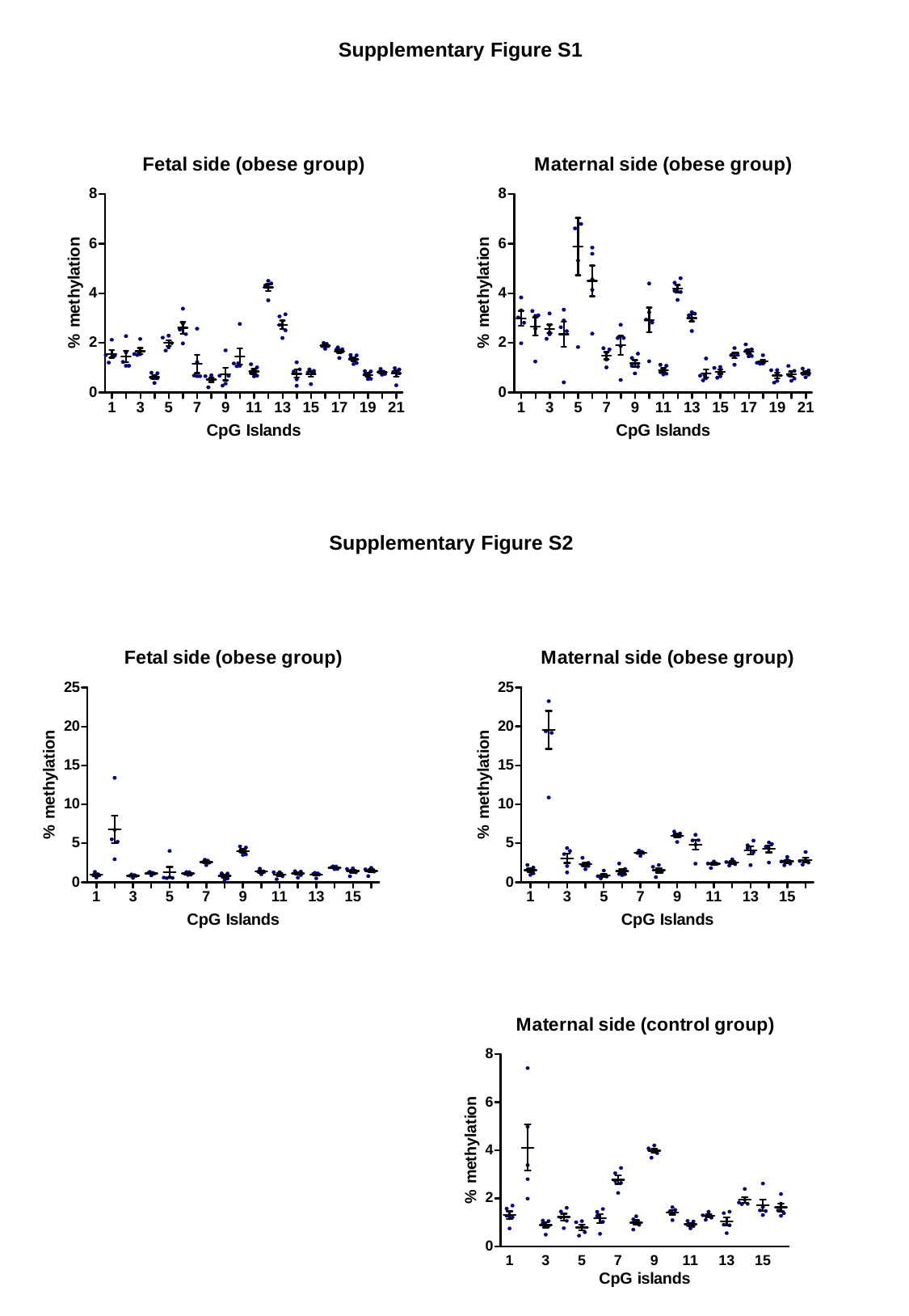

Supplementary Figure S1
Supplementary Figure S2

Supplement: Supplementary file 1 — Figure S1. DNA methylation in the promoter region of the ADIPOR1 gene in obese placenta. The methylation pattern in the ADIPOR1 promoter on the fetal and maternal sides of third-trimester placental biopsies from the obese group. The data are quoted as the mean ± SEM. *: p < 0.05 for #5 in a Wilcoxon test. Maternal side vs. fetal side in the obese group. Figure S2. DNA methylation in the promoter region of the ADIPOR2 gene in obese placenta. The methylation pattern in the ADIPOR1 promoter on the fetal and maternal sides of third-trimester placental biopsies from the obese group. The data are quoted as the mean ± SEM. *: p < 0.05 for #2 in a Wilcoxon test on Maternal side vs. fetal side in the obese group. **: p < 0.01 for #2 in a Mann-Whitney test. Maternal side in the obese group vs. the control group. (PPTX 180 kb) [file 13148_2019_612_MOESM1_ESM.pptx]
